# Prevalence of Obesity and Cardiovascular Risk Factors Among Type 2 Diabetes Mellitus Patients in Al-Khobar, Saudi Arabia

**DOI:** 10.7759/cureus.30539

**Published:** 2022-10-21

**Authors:** Noor-Ahmed Jatoi, Reem M Al-Qassab, Fatimah H Al Salem, Fatimah M Al Muzayan, Razan Z AlShammari

**Affiliations:** 1 Internal Medicine, Vascular Medicine, Imam Abdulrahman bin Faisal University, King Fahd University Hospital, Al Khobar, SAU; 2 Collage of Medicine, Imam Abdulrahman bin Faisal University, Dammam, SAU; 3 College of Medicine, Imam Abdulrahman bin Faisal University, Dammam, SAU

**Keywords:** type 2 diabetic mellitus (t2dm), metabolic health disorder, cardiovascular risk factors (cvrf), blood pressure (bp), smoking, dyslipidemia, physical activity level, obesity and overweight, waist to hip ratio (whr), body mass index (bmi)

## Abstract

Background: The prevalence of obesity has grown significantly worldwide. It is considered a major cardiovascular risk factor among type II diabetes mellitus (T2DM) patients.

Objectives: The main objective of this study is to determine the prevalence of obesity in patients with T2DM at King Fahd University Hospital (KFUH), Al-Khobar, and to assess the relationship between T2DM and cardiovascular risk factors with body mass index (BMI) and waist to hip ratio (WHR).

Methods: A retrospective, cross-sectional study, included T2DM patients from the Internal Medicine department at KFHU. The investigators recorded patient demographics (age and gender), weight (kg), height (cm), body mass index (Kg/m^2^), waist and hip circumference (cm), smoking status, physical activity, blood pressure measurements (mmHg) and laboratory results of fasting blood glucose (FBG), glycated haemoglobin (HbA1c) and lipid profile.

Results: Among 346 patients, the prevalence of obesity and overweight was 62.4% and 27.2%, respectively. The relationship between BMI and demographic data including age and gender was statistically significant (P<0.05). The correlation between the BMI with cardiovascular risk factors including smoking, physical activity and WHR found to be statistically significant (P<0.05).

Conclusion: Our study showed that obesity and overweight affect 89.6% of patients with T2DM. Therefore, it is important to take into consideration weight control strategies to effectively manage diabetic patients.

## Introduction

In recent decades, the prevalence of obesity has grown significantly around the world. Obesity could be defined as the excessive accumulation of fat in different body tissues which lead to adverse health consequences. In 2016, statistics from World Health Organization (WHO) reported that obesity affects approximately 13% of the world’s adult population [1]. In Saudi Arabia, based on the World Health Survey (WHS), prevalence of obesity and overweight were 20% and 38% respectively [2]. In fact, 88% of patients with type 2 diabetes mellitus (T2DM) were found to be overweight and obese in a study conducted in India [3]. The number of diabetic patients is expected to increase globally. By 2045 diabetes will affect over 400 million people aged 20 to 79 in the United States (US) and around a billion worldwide [4].

Obesity and overweight are measured by body mass index (BMI). However, BMI is not an accurate way to measure fat distribution in the body. While waist to hip ratio (WHR) is usually used to determine intra-abdominal and visceral fat as it is a major risk factor for cardiovascular diseases in patients withT2DM [5].

Central obesity is a feature of metabolic syndrome which is defined as having ≥ three of the following: blood pressure (BP) ≥ 130/85 mmHg, fasting blood glucose (FBG) ≥ 100 mg/dL, levels of triglyceride (TG) ≥ 150 mg/dL, high-density lipoprotein (HDL) < 50 mg/dL in women or < 40 mg/dL in men, and waist circumference ≥ 88 cm in women or ≥ 102 cm in men [6]. Patients with T2DM have a strong risk to develop metabolic syndrome and consequently a higher risk of heart disease [7].

To our knowledge, few national publications have explicitly addressed the effect of obesity on cardiovascular risk factors among T2DM patients in tertiary care hospitals. Thus, this study aimed to find the prevalence of obesity in patients with T2DM and to assess the association between BMI and WHR with the cardiovascular risk factors including, smoking, physical activity, FBG, glycated haemoglobin (HbA1c) and lipid profile.

## Materials and methods

Study population

An observational, cross-sectional study was conducted on 346 patients with T2DM. The target population was all patients with T2DM ages greater than 18 years who attended either a diabetic, endocrine, or internal medicine clinic at King Fahd University Hospital (KFUH), Al-Khobar, Saudi Arabia, from January 2015 to December 2019. Patients with diabetes mellitus type 1, younger than 18 years, and pregnant women were excluded. The minimum calculated sample size was 177 patients (using the formula N=Z2*P(1-P)ld2) at a 95% confidential interval and a 5% margin of error. 

Data collection

We collected demographics: age and gender; anthropometric measurements: weight (kg), height (cm), waist circumference (cm), hip circumference (cm), and waist-to-hip ratio (WHR); cardiovascular risk factors: smoking status, physical activity level, heart rate (HR), systolic BP, diastolic blood pressure, pulse pressure (PP) and mean arterial pressure (MAP = DP+1/3[Systolic BP-Diastolic BP]); laboratory results: HbA1c, FBG, lipid profile: low-density lipoprotein (LDL), HDL, total cholesterol and TG. Patients' incomplete data before the hospital's electronic Quadra-Med system was completed from their previous medical charts and incomplete smoking and physical activity information was collected when they visited the outpatient clinic.

The dependent variables were BMI and WHR. BMI was calculated using weight (kg) and height (cm) according to the formula (weight (kg) / height (m)2). Weight was classified according to BMI as underweight (<18.5 kg/m^2^), normal weight (18.5-24.9 kg/m^2^), overweight (≥25-29.9 kg/m^2^), obesity (≥30.00 kg/m^2^) [8]. WHR was calculated by dividing the waist circumference (cm) and hip circumference (cm). WHR is classified into low cardiovascular risk (≤ 0.8 in females and ≤0.95 in males), moderate cardiovascular risk (0.81-0.85 in females and 0.96-1.0 in males), and high cardiovascular risk (≥0.86 in females and ≥1.0 in males) [9]. Activity level was classified into active >150 minutes/week, moderate active >100 and <150 minutes/week, mild active > 50 and < 100 minutes/week, and inactive [10]. FBG was classified into <80 mg/dL, from 81 to 130 mg/dL and more than 130 mg/dL [11]. HbA1c was classified into controlled <7% and uncontrolled ≥7% in treated T2DM patients [12,13]. Lipid profile classified as total cholesterol: normal <200 mg/dL, borderline 200-239 and impaired ≥ 240 mg/dL. LDL: normal ≤ 129 mg/dL, borderline 130-159 mg/dL and ≥ 160. HDL: Low <40 mg/dL, normal 40-60 mg/dL, high >60 mg/dL. TG: normal <150 mg/dL, borderline 150-199 mg/dL, high ≥ 200 mg/dL [14].

The study was held in accordance with the World Medical Association Declaration of Helsinki 1975 (revised in 2000). Ethical approval for this study was obtained from the Institutional Review Board (IRB-UG-2021-01-345) of Imam Abdul-Rahman Bin Faisal University (IAU) in Saudi Arabia.

Data analysis

The data were organized, tabulated, and analyzed using the standard computer program IBM Statistical Package for the Social Sciences (SPSS) Statistics for Mac, version 22 (IBM Corp., Armonk, NY, USA). Descriptive analyses were obtained by counts, percentages, and mean ± standard deviation (SD). In addition, potential associations were tested through the Chi-squared test, Pearson Chi-Squared test, bivariate and multivariate analysis to be of statistical significance at P<0.05 to assess the association between tested variables.

## Results

Participants' demographics and BMI status are shown in Table 1.

**Table 1:** Demographic and clinical characteristics of the study population (n 346).

Characteristic	Mean ± SD or (%)
Age (years)	58.1 ± 12.5
Male: Female (%)	(57:43)
Height (cm)	163 ± 9.4
Weight (kg)	84.4 ± 17.4
Body mass index (kg/m^2^)	31.8 ± 6.3
Healthy (18.5-24.9)	(10.4)
Overweight (25-29.9)	(27.2)
Obese (30 and more)	(62.4)
Waist (cm)	109.7± 13.8
Hip (cm)	111 ± 12.7
Waist-to-hip ratio	0.99 ± 0.9
Low waist-to-hip ratio (%)	(18.4)
Moderate waist-to-hip ratio (%)	(13.0)
High waist-to-hip ratio	(68.6)
Smoking status	
Current smoker (%)	(14)
Ex-smoker (%)	(19)
Never smoke (%)	(67)
Activities level	
Very active (%)	(13.9)
Moderately active (%)	(6.4)
Mildly active (%)	(10.1)
Inactive (%)	(37.6)
Haemodynamic measurements	
Systolic blood pressure (mmHg)	136.4 ± 18
Diastolic blood pressure (mmHg)	78.7 ± 11
Pulse pressure (mmHg)	56± 17.5
Mean arterial pressure (mmHg)	94.9 ± 20.6
Heart rate (beats/minutes)	82 ± 14
Laboratory results	
Total cholesterol (mg/dL)	164.1 ± 45.1
Low density lipoprotein (mg/dL)	100.7 ± 39.5
High density lipoprotein (mg/dL)	43.4 ± 11.7
Triglycerides (mg/dL)	142 ± 78
Fasting blood sugar (mg/dL)	165 ± 73
Glycosylated hemoglobin (%)	8.6 ± 1.9
All values are presented as number sand percentage. SD = Standard deviation	

Our study showed that 62.4% of diabetic patients who came to our clinic were obese. Males were found to have a higher percentage of overweight at 68.1%, while females have higher percentages of obesity at 51.4%. There was a statistically significant association between different BMI categories and gender (P=0.000). In addition, age was statistically significant with healthy, overweight, and obese BMI status (P=0.008). Results from the bivariate analysis showed that there was no significant association between BMI and WHR status with the demographic characteristics (all P>0.05).

BMI and cardiovascular risk factors

Regarding the cardiovascular risk factors, more than half the sample size (68.6%) had elevated WHR, and 67.4% of the study population had never smoked. A statistically significant association was identified between BMI and smoking status, WHR, and physical activity level (P<0.05). Details of demographics and cardiovascular risk factors are presented in Table 2. There was a significant positive correlation between the WHR and physical activity (r=0.175 P<0.05). With regard to smoking, there was no significant positive correlation with WHR.

**Table 2:** The association between Body Mass Index (BMI) and participants’ demographic factors and cardiovascular risk factors (n 346).

Characteristic	Total n= 346 (%)	Body Mass Index (BMI)			P-value
		Healthy weight n= 36 (%)	Overweight n= 94 (%)	Obese n=216 (%)	
Demographics					
Gender					
Male	197 (56.9)	28 (77.8)	64 (68.1)	105 (48.6)	0.000*
Female	149 (43.1)	8 (22.2)	30 (31.9)	111 (51.4)	
Age					
40 years and less	34 (9.8)	9 (25.0)	4 (4.3)	21 (9.7)	0.008*
From 41 to 60 years	148 (42.8)	9 (25.0)	46 (48.9)	93 (43.1)	
61 years and above	164 (47.4)	18 (50.0)	44 (46.8)	102 (47.2)	
Cardiovascular risk factors					
Smoking status					
Current smoker	48 (13.9)	11 (30.0)	14 (15.3)	22(10.4)	0.017*
Ex-smoker	65 (18.8)	6 (16.7)	24 (25.9)	34 (15.6)	
Never	233 (67.4)	19 (53.3)	55 (58.8)	160 (74)	
Activity level					
Very Active	48 (13.9)	8 (22.2)	21 (22.3)	19 (8.8)	0.006*
Moderately active	22 (6.4)	4 (11.1)	5 (5.3)	13 (6.0)	
Mildly active	35 (10.1)	4 (11.1)	15 (16.0)	16 (7.4)	
Inactive	130 (37.6)	11 (30.6)	29 (30.9)	90 (41.7)	
Waist-hip ratio (WHR)					
Low	64 (18.4)	16 (43.5)	23 (24.2)	22 (10.2)	0.001*
Moderate	45 (13.0)	8 (21.7)	11 (12.1)	26 (11.9)	
High	237 (68.6)	12 (34.8)	60 (63.6)	168 (78)	
*Association found at 0.05 level of significant.					

Laboratory monitoring parameters

For laboratory results in Table 3, the FBG of 63% of participants exceeded 130 mg/dL with a mean FBG of 165 (SD ± 73). Seventy-eight percent of the study participants had uncontrolled HbA1c values compared to 22% who were controlled with a mean HbA1c value of 8.6% (SD ± 1.9). There was no significant association between BMI status with FBG, and HbA1c (P>0.05). However, FBG and HbA1c had a positive significant positive correlation with the WHR (r=0.165, P=0.025<0.05, r=0.164, P=0.022<0.05). In addition, the association between participants’ BMI status and their lipid profile was statistically insignificant (P>0.05), as presented in Table 3. Results from the bivariate analysis showed that there was no significant correlation between BMI status with the lipid levels (all P>0.05). Also, the WHR and lipid profile shows no significant correlation with BMI.

**Table 3:** The association between Body Mass Index (BMI) and participants’ laboratory results (n 346).

Laboratory results	Total (%) n= 346	Body Mass Index (BMI)			P-value
		Healthy weight (%) n= 36	Overweight (%) n= 94	Obesity (%) n=216	
Fasting blood glucose (FBG) (mg/dl)					
less than 80	13 (4.2)	2 (6.1)	2 (2.4)	9 (4.6)	0.887
81 to 130	102 (32.8)	11 (33.3)	28 (33.7)	63 (32.3)	
More than 130	196 (63.0)	20 (60.6)	53 (63.9)	123 (63.1)	
Glycosylated hemoglobin (HbA1C) (%)					
Controlled	71 (22.0)	8 (22.9)	19 (21.8)	44 (22.0)	0.992
Uncontrolled	251 (78.0)	27 (77.1)	68 (78.2)	156 (78.0)	
Lipid profile					
Total Cholesterol (mg/dl)					
Normal: less than 200	257 (81.1)	27 (87.1)	67 (77.9)	163 (81.5)	0.393
Borderline: 200 – 239	38 (12.0)	1 (3.2)	12 (14.0)	25 (12.5)	
Impaired: 240 and more	22 (6.9)	3 (9.7)	7 (8.1)	12 (6.0)	
Low density lipoprotein (LDL) (mg/dl)					
Normal: 129 and less	252 (79.5)	27 (87.1)	68 (79.1)	157 (78.5)	0.442
Borderline: 130-159	34 (10.7)	3 (9.7)	7 (8.1)	24 (12.0)	
High: 160 and more	31 (9.8)	1 (3.2)	11 (12.8)	19 (9.5)	
High density lipoprotein (HDL) (mg/dl)					
low if less than 40	137 (43.2)	15 (48.4)	30 (34.9)	92 (46.0)	0.274
Normal 40-60m	140 (44.2)	14 (45.2)	45 (52.3)	81 (40.5)	
High if more than 60	40 (12.6)	2 (6.5)	11 (12.8)	27 (13.5)	
Triglyceride (TG) (mg/dl)					
Normal: less than 150	205 (64.7)	21 (67.7)	61 (70.9)	123 (61.5)	0.645
Borderline: 150 – 199	55 (17.4)	5 (16.1)	12 (14.0)	38 (19.0)	
High: 200 and more	57 (18.0)	5 (16.1)	13 (15.1)	39 (19.5)	
*Association found at 0.05 level of significant.					

## Discussion

Our study was conducted to determine the prevalence of obesity in T2DM patients and to explore the association between BMI and WHR with cardiovascular risk factors. The study results revealed a high prevalence of both obesity and overweight in T2DM patients of 89.6%, among which 62.4% were obese and 27.2% were overweight. In contrast, healthy weight accounted for only 9.2%.

Similar findings were observed in a study conducted in Saudi Arabia by Alshahrani et al. (2021), where 57.8% of diabetic patients were obese and 27.9% were overweight [15]. However, other local studies showed less prevalence of obesity in T2DM patients, 39.9%, and 38.3% in Khobar and Jeddah, respectively [16,17]. The variance in the results could be attributable to sample size discrepancies.

Moreover, in a study conducted in the United Kingdom (UK) by Daousi et al. (2006) the prevalence of both overweight and obesity in T2DM patients was found to be 86% overall, however, 52% of them were obese and 34% were overweight [18]. The obvious increase in the prevalence could be due to the fact that obesity is linked with insulin resistance which strongly increases the risk of diabetes [19]. Furthermore, lifestyle factors include unhealthy dietary habits and high caloric intake, as well as a sedentary lifestyle and physical inactivity.

The relationship between BMI and age was statistically significant in our study. The age range of 61 years and above had the highest percentage of obesity (47.2%). Our finding is consistent with another study conducted in Bisha, Saudi Arabia [15]. On the other hand, several studies stated that patients older than 60 have the least prevalence of obesity [20,21]. The disparity in results could be due to genetic differences.

Current study results revealed a significant association between BMI and gender. Obesity was higher in females (51.4%), whereas overweight was higher in males (68.1%). This result is in line with numerous studies [16-18,21,22]. However, other studies found that both obesity and overweight are more common in females [20,23,24]. The difference in the prevalence of obesity and overweight between males and females could be attributed to many factors. Physiological and hormonal changes peri and post-menopausal make females more susceptible to weight gain, in addition to the region-specific norms [25].

In our study, the correlation between BMI and WHR was statistically significant. 68.6% of T2DM patients had high WHR and only 13% had moderate WHR. This finding is similar to previous studies, as diabetic patients have a higher percentage of intraabdominal fat, therefore higher WHR [26-28]. However, no studies assessed the prevalence and the association between BMI and cardiovascular risk factors in T2DM patients in Saudi Arabia Al-Khobar (KFUH). 

Regarding smoking habits, although most of the study participants never smoked (67.4%), the majority of them were found to be obese. On the other hand, most of the ex-smokers are overweight and current smokers have a healthy weight with a statistically significant relation. This could be attributed to the appetite suppression effect of nicotine [29]. Smoking is usually associated with high levels of nicotine in the blood. Nicotine reduces the uptake of glucose by body cells leading to hyperglycemia and insulin resistance [30]. Therefore, smokers are more prone to develop T2DM compared to nonsmokers [31-33].

Physical inactivity is an additional cardiovascular risk factor found in our study which has a significant correlation with BMI. The majority of inactive participants are obese (41.7%). In fact, physical inactivity can lead to obesity which is an independent risk factor for T2DM [30].

Current study results revealed that the percentage of controlled and uncontrolled HbA1c in obese individuals with T2DM were 22% and 78% respectively. However, the association between BMI categories and HbA1c levels was statistically insignificant. The high percentage of uncontrolled HbA1c in obese individuals was attributed to several factors that affect glycemic control other than BMI. Other factors include the long duration of diabetes, combined oral antidiabetic and insulin therapy, noncompliance to medication, poor dietary regimen, and physical inactivity [34-35].

Our study findings are inconsistent with previous studies. According to Bae et al. (2016) being overweight or obese is associated with a higher probability of having uncontrolled HbA1c ≥7%. In comparison, normal-weight individuals tend to have controlled levels of HbA1C <7% [36]. The previous findings are in parallel with another study by Weng et al. (2017) which stated that as the BMI status increases the percentage of uncontrolled HbA1c increases, and the percentage of controlled HbA1c decreases [37]. In addition, obese individuals were found to have worse glycemic control in comparison with overweight individuals [35,37]. The statistically insignificant association between BMI and HbA1c levels in our study might be due to the small sample size compared with the previous studies [36,38].

In this study, the association between BMI of healthy weight, overweight and obese T2DM with lipid profile was higher but p value was not significant. Our results are in accordance with a study in Pakistan by Hussain et al. (2019), where total cholesterol, TG, and LDL showed no correlation with BMI [33]. In addition, another study in Nepal by Bansal et al. (2018), revealed that BMI had a correlation with LDL, HDL, and other lipid parameters, however, all of these correlations were statistically insignificant [39].

In our study, the association between WHR and demographic data was not statistically significant. However, WHR was significantly higher in old patients aged 40 and more and female gender in a study conducted in Ghana by Mogre et al. (2014) [40].

Our study revealed an inverse correlation between WHR and physical activity. With regards to smoking, no significant positive correlation was found with WHR. Further studies are required to address the association between WHR with physical activity and smoking as no studies are currently available. However, in the study conducted in Ghana by Mogre et al. [40], WHR was significantly higher in those aged 40 or older and in females. For every 10% increase in WHR risk of getting T2DM increases by 28% [41].

This study showed a positive significant association between WHR with FBG and HbA1c. However, there are no previous studies about the association between these variables in T2DM patients. The current study results showed that there was no significant association between WHR and lipid profile. However, these are inconsistent with previous studies as Biadgo et al. (2017) revealed a statistically significant positive association between WHR with total cholesterol, LDL, and TG in T2DM patients [42]. According to Himabindu et al. (2013) there was a statistically significant inversed association between WHR and HDL [43]. In addition, Choi et al. (2012) study results showed that there was a statistically significant association between WHR with total cholesterol and LDL in T2DM male patients [44].

Our study has several limitations which consequently affect the results. The sample size is considered small and limited in comparison with different studies conducted at national and international levels. This could be due to the limited time of the data collection as well as the missing data of some patients in the hospital system (Quadra-Med). It is inapplicable to generalize the study results to all Saudi population because the data was obtained from a single hospital system and the sample size is small. Moreover, primary care physicians and endocrinologists should encourage their patients to have applicable weight reduction strategies in order to adjust their cardiovascular risk factors as well as obesity-related complications. We recommend conducting similar studies among a larger sample size at multiple centers to have more representative results of the Saudi population.

## Conclusions

The study found a prevalence of both overweight and obesity among T2DM patients at KFUH, Saudi Arabia. Higher prevalence was found among the elderly, females, non-smokers, physically inactive individuals, and patients with high WHR. According to our study results, strict lifestyle modifications, healthy dietary habits and increasing level of physical activity are important to decrease obesity-related complications. Moreover, healthcare providers should screen them regularly for the cardiovascular risk factors.

## References

[1] (2022). Obesity and Overweight. World Health Organization.

[2] (2022). World Health Survey Saudi Arabia. https://www.moh.gov.sa/en/Ministry/Statistics/Population-Health-Indicators/Documents/World-Health-Survey-Saudi-Arabia.pdf.

[3] Vasanthakumar J, Kambar S (2020). Prevalence of obesity among type 2 diabetes mellitus patients in urban areas of Belagavi. Indian J Heal Sci Biomed Res.

[4] Pandit R, Pandit T, Goyal L, Ajmera K (2022). A review of national level guidelines for risk management of cardiovascular and diabetic disease. Cureus.

[5] Shirafkan A, Marjani A (2011). Prevalence of obesity among type 2 diabetes mellitus in Gorgan (South East of Caspian Sea). Iran J Chinese Clin Med.

[6] Swarup S, Goyal A, Grigorova Y, Zeltser R (2020). Metabolic Syndrome. StatPearls - NCBI Bookshelf.

[7] LeRoith D (2008). Hyperglycemia, hypertension, and dyslipidemia in type 2 diabetes mellitus: goals for diabetes management. Clin Cornerstone.

[8] (2022). Classification of Overweight and Obesity by BMI, Waist Circumference, and Associated Disease Risks. https://www.nhlbi.nih.gov/health/educational/lose_wt/BMI/bmi_dis.htm.

[9] (2022). Waist Circumference and Waist-Hip ratio: report of a WHO Expert Consultation. https://www.who.int/publications/i/item/9789241501491.

[10] Okely AD, Kontsevaya A, Ng J, Abdeta C (2021). 2020 WHO guidelines on physical activity and sedentary behavior. Sports Med Health Sci.

[11] (2022). Blood Sugar Level Ranges. https://www.diabetes.co.uk/diabetes_care/blood-sugar-level-ranges.html.

[12] (2022). Saudi National Diabetes Center (SNDC). https://shc.gov.sa/Arabic/Documents/SDCPGuidelines.pdf.

[13] (2022). Guide to HbA1c. https://www.diabetes.co.uk/category/blood-glucose/.

[14] (2001). Executive summary of the third report of the National Cholesterol Education Program (NCEP) expert panel on detection, evaluation, and treatment of high blood cholesterol in adults (adult treatment panel III). JAMA.

[15] AlShahrani MS (2021). Prevalence of obesity and overweight among type 2 diabetic patients in Bisha, Saudi Arabia. J Family Med Prim Care.

[16] Mugharbel KM, Al-Mansouri MA (2003). Prevalence of obesity among type 2 diabetic patients in Al-khobar primary health care centers. J Family Community Med.

[17] Bakhotmah BA (2013). Prevalence of obesity among type 2 diabetic patients: non-smokers housewives are the most affected in Jeddah, Saudi Arabia. Open J Endocr Metab Dis.

[18] Daousi C, Casson IF, Gill GV, MacFarlane IA, Wilding JP, Pinkney JH (2006). Prevalence of obesity in type 2 diabetes in secondary care: association with cardiovascular risk factors. Postgrad Med J.

[19] Al-Goblan AS, Al-Alfi MA, Khan MZ (2014). Mechanism linking diabetes mellitus and obesity. Diabetes Metab Syndr Obes.

[20] Damian DJ, Kimaro K, Mselle G, Kaaya R, Lyaruu I (2017). Prevalence of overweight and obesity among type 2 diabetic patients attending diabetes clinics in northern Tanzania. BMC Res Notes.

[21] Al-Sharafi BA, Gunaid AA (2014). Prevalence of obesity in patients with type 2 diabetes mellitus in Yemen. Int J Endocrinol Metab.

[22] Alqurashi KA, Aljabri KS, Bokhari SA (2011). Prevalence of diabetes mellitus in a Saudi community. Ann Saudi Med.

[23] Tino S, Mayanja BN, Mubiru MC, Eling E, Ddumba E, Kaleebu P, Nyirenda M (2020). Prevalence and factors associated with overweight and obesity among patients with type 2 diabetes mellitus in Uganda-a descriptive retrospective study. BMJ Open.

[24] Basukala A, Sharma M, Pandeya A (2014). Prevalence of overweight and obesity among patients with type 2 diabetes mellitus in Kathmandu. Sky J Microbiol Res.

[25] Favieri F, Forte G, Pazzaglia M, Chen EY, Casagrande M (2022). High-level executive functions: a possible role of sex and weight condition in planning and decision-making performances. Brain Sci.

[26] Bellou V, Belbasis L, Tzoulaki I, Evangelou E (2018). Risk factors for type 2 diabetes mellitus: an exposure-wide umbrella review of meta-analyses. PLoS One.

[27] Kahn BB, Flier JS (2000). Obesity and insulin resistance. J Clin Invest.

[28] De K (2017). Waist circumference, waist-hip ratio and body mass index in assessing nutritional status and central obesity of adolescent. Glob J Archaeol Anthropol.

[29] Stadler M, Tomann L, Storka A (2014). Effects of smoking cessation on β-cell function, insulin sensitivity, body weight, and appetite. Eur J Endocrinol.

[30] Ismail L, Materwala H, Al Kaabi J (2021). Association of risk factors with type 2 diabetes: a systematic review. Comput Struct Biotechnol J.

[31] Yuan S, Larsson SC (2020). An atlas on risk factors for type 2 diabetes: a wide-angled Mendelian randomisation study. Diabetologia.

[32] Fatimah RN (2015). Diabetes mellitus type 2. Fak Kedokt Univ Lampung.

[33] Cena H, Fonte ML, Turconi G (2011). Relationship between smoking and metabolic syndrome. Nutr Rev.

[34] Khattab M, Khader YS, Al-Khawaldeh A, Ajlouni K (2010). Factors associated with poor glycemic control among patients with type 2 diabetes. J Diabetes Complications.

[35] Fekadu G, Bula K, Bayisa G, Turi E, Tolossa T, Kasaye HK (2019). Challenges and factors associated with poor glycemic control among type 2 diabetes mellitus patients at nekemte referral hospital, Western Ethiopia. J Multidiscip Healthc.

[36] Bae JP, Lage MJ, Mo D, Nelson DR, Hoogwerf BJ (2016). Obesity and glycemic control in patients with diabetes mellitus: analysis of physician electronic health records in the US from 2009-2011. J Diabetes Complications.

[37] Weng W, Tian Y, Kimball ES, Kong SX, Bouchard J, Hobbs TM, Sakurada B (2017). Treatment patterns and clinical characteristics of patients with type 2 diabetes mellitus according to body mass index: findings from an electronic medical records database. BMJ Open Diabetes Res Care.

[38] Boye KS, Lage MJ, Thieu V, Shinde S, Dhamija S, Bae JP (2021). Obesity and glycemic control among people with type 2 diabetes in the United States: a retrospective cohort study using insurance claims data. J Diabetes Complications.

[39] Bansal P, Upadhyay HP (2018). Correlation between body mass index and lipid profile in a diabetic population of central Nepal. J Coll Med Sci.

[40] Mogre V, Abedandi R, Salifu ZS (2014). Correlates and predictors of increasing waist circumference in patients with type 2 diabetes mellitus: a cross-sectional study. Int Sch Res Notices.

[41] Jafari-Koshki T, Mansourian M, Hosseini SM, Amini M (2016). Association of waist and hip circumference and waist-hip ratio with type 2 diabetes risk in first-degree relatives. J Diabetes Complications.

[42] Biadgo B, Abebe SM, Baynes HW, Yesuf M, Alemu A, Abebe M (2017). Correlation between serum lipid profile with anthropometric and clinical variables in patients with type 2 diabetes mellitus. Ethiop J Health Sci.

[43] Himabindu Y, Sriharibabu M, Alekhya K, Saisumanth K, Lakshmanrao N, Komali K (2013). Correlations between anthropometry and lipid profile in type 2 diabetics. Indian J Endocrinol Metab.

[44] Choi S (2011). Anthropometric measures and lipid coronary heart disease risk factors in Korean immigrants with type 2 diabetes. J Cardiovasc Nurs.

